# Multifunctional Microtubule-Associated Proteins in Plants

**DOI:** 10.3389/fpls.2016.00474

**Published:** 2016-04-21

**Authors:** Jana Krtková, Martina Benáková, Kateřina Schwarzerová

**Affiliations:** ^1^Department of Biology, University of WashingtonSeattle, WA, USA; ^2^Katerina Schwarzerová Lab, Department of Experimental Plant Biology, Faculty of Science, Charles University in PraguePrague, Czech Republic; ^3^Department of Biology, Faculty of Science, University of Hradec KrálovéRokitanského, Czech Republic

**Keywords:** multifunctional MAP, MAP, microtubules, tubulin, plants

## Abstract

Microtubules (MTs) are involved in key processes in plant cells, including cell division, growth and development. MT-interacting proteins modulate MT dynamics and organization, mediating functional and structural interaction of MTs with other cell structures. In addition to conventional microtubule-associated proteins (MAPs) in plants, there are many other MT-binding proteins whose primary function is not related to the regulation of MTs. This review focuses on enzymes, chaperones, or proteins primarily involved in other processes that also bind to MTs. The MT-binding activity of these multifunctional MAPs is often performed only under specific environmental or physiological conditions, or they bind to MTs only as components of a larger MT-binding protein complex. The involvement of multifunctional MAPs in these interactions may underlie physiological and morphogenetic events, e.g., under specific environmental or developmental conditions. Uncovering MT-binding activity of these proteins, although challenging, may contribute to understanding of the novel functions of the MT cytoskeleton in plant biological processes.

## MAPs and their role in plant cells

Traditional microtubule-associated proteins (MAPs) are typically conserved in eukaryotes. However, plants possess a set of MAPs specific to plant morphology and physiology (Gardiner, 2013). A fundamental feature of MAPs is their interaction with MTs through a MT-binding domain that is manifested in MT organization, dynamics or cellular transport, which influences plant morphogenesis. The localization of MAPs in the cell is well described mainly because of their close association with MT structures. Consequently, MAPs are direct MT-interactors and their function is dependent on their MT-binding activity (Buschmann and Lloyd, 2008). MAPs are motor proteins that utilize MTs as tracks to transport cargo such as kinesins. Structural MAPs or severing proteins such as MAP65 and katanin bind, bundle or cleave MTs, and therefore, are involved in MT organization. MT dynamics, on the other hand, is influenced by MT +tip associated proteins (+TIPs), such as, e.g., CLASP, EB1, etc., through their binding and interactions at the +end of growing MT. Conventional MAPs have been reviewed in several outstanding reviews (for instance Lloyd and Hussey, 2001; Akhmanova and Steinmetz, 2008; Sedbrook and Kaloriti, 2008; Gardiner, 2013; Hamada, 2014; Hashimoto, 2015; Li et al., 2015) and will not be discussed here. MAPs identified in plants are listed in the Table 1.

**Table 1:** **List of MAPs described in plants**.

**MAP**	**Characterized in**	**References**	**Notes**
**MAP65**			
MAP65-1	*Arabidopsis thaliana*	Jiang and Sonobe, 1993; Smertenko et al., 2004; Van Damme et al., 2004	
MAP65-2	*Arabidopsis thaliana*	Li et al., 2009	
MAP65-3/PLEIADE	*Arabidopsis thaliana*	Muller et al., 2004	
MAP65-4	*Arabidopsis thaliana*	Van Damme et al., 2004	
MAP65-5		Gaillard et al., 2008; Smertenko et al., 2008	
MAP65-6		Mao et al., 2005	
MAP65-7		Theologis et al., 2000	Found *in silico* in *Arabidopsis*
MAP65-8	*Arabidopsis thaliana*	Smertenko et al., 2008	Does not associate with MT
MAP65-9	*Arabidopsis thaliana*	Smertenko et al., 2008	Pollen, does not associate with MT
**MT** +**END BINDING PROTEINS**			
EB1	*Arabidopsis thaliana*	Chan et al., 2003; Mathur et al., 2003	
CLASP	*Arabidopsis thaliana*	Ambrose et al., 2007; Kirik et al., 2007	
MOR/GEM1	*Arabidopsis thaliana*	Whittington et al., 2001	
TMBP200	*Nicotiana tabacum* (BY-2)	Yasuhara et al., 2002; Hamada et al., 2004	
AUG8	*Arabidopsis thaliana*	Cao et al., 2013	
**KINESINS AND KINESIN-LIKE PROTEINS**			
ATK5	*Arabidopsis thaliana*	Ambrose et al., 2005	Binds also to MT + ends
KCH1 (kinesin with calponin homology 1)	*Gossypium hirsutum, Oryza sativa*	Preuss et al., 2004; Frey et al., 2009	
KCH2 (kinesin with calponin homology 2)	*Gossypium hirsutum*	Xu et al., 2009	
O12	*Oryza sativa*	Umezu et al., 2011	
kinesin 13-A	*Nicotiana tabacum*	Wei et al., 2005	
KCBP/ZWICKEL	*Arabidopsis thaliana*	Krishnakumar and Oppenheimer, 1999	
TBK5	*Nicotiana tabacum*	Matsui et al., 2001	
AtPAKRP1	*Arabidopsis thaliana, Oryza sativa*	Lee and Liu, 2000	
DcKRP120-2	*Daucus carota*	Barroso et al., 2000	
TKRP125	*Nicotiana tabacum*	Asada et al., 1997	
KINID1	*Physcomitrella patens*	Hiwatashi et al., 2014	
KatA	*Arabidopsis thaliana*	Liu B. et al., 1996	
KatD	*Arabidopsis thaliana*	Tamura et al., 1999	
**OTHER PROTEINS**			
TANGLED 1	*Zea mays*	Smith et al., 2001	
p60 katanin subunit (AtKSS, AtKN1)	*Arabidopsis thaliana*	Burk et al., 2001	
p80 katanin subunit	*Arabidopsis thaliana*	Bouquin et al., 2003	
RUNKEL (RUK)	*Arabidopsis thaliana*	Krupnova et al., 2009	
Spc98p	*Arabidopsis thaliana*	Erhardt et al., 2002	
BPP1	*Arabidopsis thaliana*	Hamada et al., 2013	
NEDD1	*Arabidopsis thaliana*	Zeng et al., 2009	Acts as an anchoring factor of γ-tubulin complex, decorates spindle MTs preferentially toward theirs minus ends
**PLANT SPECIFIC MAPS**			
MAP190	*Nicotiana tabacum* (BY-2)	Igarashi et al., 2000	
MAP70 -1	*Arabidopsis thaliana*	Korolev et al., 2005; Pesquet et al., 2010	
MAP70 -2	*Arabidopsis thaliana*	Korolev et al., 2005	
MAP70 -3	*Arabidopsis thaliana*	Korolev et al., 2005	
MAP70 -4	*Arabidopsis thaliana*	Korolev et al., 2005	
MAP70 -5	*Arabidopsis thaliana*	Korolev et al., 2005, 2007	
SPR1	*Arabidopsis thaliana*	Nakajima et al., 2004; Sedbrook, 2004	
SPR2	*Arabidopsis thaliana*	Furutani et al., 2000	
SB401	*Solanum berthaultii*	Huang et al., 2007	
SBgLR	*Nicotiana tabacum*	Liu et al., 2013	Potato pollen-specific protein
Atg8	*Arabidopsis thaliana*	Ketelaar et al., 2004	Homolog of autophagy protein
AtMPB2C	*Arabidopsis thaliana*	Ruggenthaler et al., 2009	Homolog of MPB2C, involved in the alignment of cortical MT
MDP40	*Arabidopsis thaliana*	Wang et al., 2012	Regulator of hypocotyl cell elongation
WVD/WDL family	*Arabidopsis thaliana*	Perrin et al., 2007	
AIR9	*Arabidopsis thaliana*	Buschmann et al., 2006	

## Multifunctional MAPs

Additionally to numerous MAPs, MTs probably interact with other “fine tuning” factors that are most likely required for microtubular functions as well. The function of these proteins is not primarily related to MT-binding, but they may bind to MTs only under specific conditions. The cytoskeleton is a three-dimensional dynamic structure that can be thought of as a framework for compartmentalization of cytosolic regions. Binding of proteins to this scaffold may increase the efficiency of cellular processes by bringing interacting molecules together in place and time. In this case, MTs may function as a passive structure. Additionally, metabolic enzymes such as GAPDH have been shown to modulate MT cytoskeleton (Sirover, 1999). This suggests that multiple proteins may interact with MTs to integrate signaling pathways and the reorganization of microtubules. Along with MT-binding, these proteins perform other, MT-unrelated functions. For this review, we will refer to these proteins as multifunctional MAPs as a way to distinguish them from MAPs that exclusively regulate MT structure and dynamics.

It is possible that multifunctional MAPs lack a well-defined MT-binding domain, are members of larger protein complexes and, therefore, are not found using database-based sequence similarity searches for MAPs. Instead, biochemical methods are required for their detection. The existence of a large number of proteins with known MT-unrelated functions that unexpectedly interacted with MT cytoskeleton has been documented in proteomic searches for MT-interacting proteins (Chuong et al., 2004; Korolev et al., 2005; Hamada et al., 2013; Derbyshire et al., 2015). In these experiments, tens to hundreds of cytosolic proteins interacting with tubulin or MTs were identified. However, conventional MAPs represented only a minor portion of the total MT-interacting fraction. For example, Chuong et al. (2004) used tubulin-affinity chromatography to identify a set of proteins interacting with tubulin. Only 6% of proteins were predicted as known MAPs in this protein group. Similarly, liquid chromatography-tandem mass spectrometry of MAPs-enriched fraction from *Arabidopsis* suspension cells was used by Hamada et al. (2013) to identify hundreds of proteins. Replication, transcription and translation-associated proteins were enriched here as well (Hamada et al., 2013). Derbyshire et al. (2015) performed a MT-pull-down protein search for MT-interacting proteins exhibiting differential accumulation during tracheary element differentiation; only 3% of proteins were classified as known MAPs (Derbyshire et al., 2015).

On the other hand, the presence of the protein in MAP enriched fraction does not always indicate its direct association with MTs. For each putative multifunctional MAP, the association with MTs or tubulin indicated by biochemical isolation needs to be tested by other methods. In contrast to MAPs, investigating the role of multifunctional MAPs associated with MTs is usually challenging. Multifunctional MAPs often cannot perform their MT-related function alone; their affinity to MTs may be dependent on factors such as upstream or feedback regulations, may be phospho- or ligand-dependent or may be of short duration, e.g., as for Hsp90 (Krtkova et al., 2012). Here we review plant proteins repeatedly found to associate with MTs whose primary function is distinct from MT-binding (Table 2). If possible, we provide a short description of their physiological function in the association with MTs.

**Table 2:** **List of multifunctional MAPs described in plants**.

**MAP**	**Characterized in**	**References**	**Notes**
**ENZYMES OR CHAPERONS**			
GAPDH	Mammalian cells	Sirover, 1999; Tisdale et al., 2009	
Glycolytic enzymes: lactate-dehydrogenase, pyruvate kinase, aldolase and during specific conditions also for glucose-6-phosphate isomerase and phosphoglycerate-kinase		Walsh et al., 1989	
Hsp70	*Arabidopsis thaliana*	Ho et al., 2009	Also involved in signaling
Hsp90		Koyasu et al., 1986; Sanchez et al., 1988; Williams and Nelsen, 1997; Freudenreich and Nick, 1998; Petrasek et al., 1998; Pratt et al., 1999; Lange et al., 2000; de Carcer et al., 2001; Harrell et al., 2002; Wegele et al., 2004; Glover, 2005; Basto et al., 2007; Weis et al., 2010; Krtkova et al., 2012	Also involved in signaling
Plant chaperone CCT	*Nicotiana tabacum*	Nick et al., 2000	
EF1α	*Daucus carota*	Durso and Cyr, 1994	
EF-2	*Arabidopsis thaliana*, suspension cells	Chuong et al., 2004	
PLDδ	*Nicotiana tabacum*	Gardiner et al., 2001	Also involved in signaling
THO2	*Nicotiana tabacum*	Hamada et al., 2009	Putative RNA-processing THO2 relative protein
**PROTEINS INTERACTING WITH OTHER CELL STRUCTURES**			
**Actin Binding Proteins**			
FH4	*Arabidopsis thaliana*	Deeks et al., 2010	Also involved in signaling
FH14	*Arabidopsis thaliana*	Li et al., 2010	aLso involved in signaling
FH1	*Arabidopsis thaliana*	Rosero et al., 2013	Also involved in signaling
ARPC2	*Nicotiana tabacum*	Havelková et al., 2015	
**Proteins Involved in Signaling**			
PCaP2 (MAP18)	*Arabidopsis thaliana*	Wang et al., 2007; Kato et al., 2010	
MDP25 (PCaP1)	*Arabidopsis thaliana*	Li et al., 2011	PCAP1, MT destabilizing protein
MIDD1	*Arabidopsis thaliana*	Oda et al., 2010	MT-end tracking protein

## Enzymes and chaperones

Chaperone proteins and metabolic enzymes have been repeatedly found in MT-interacting protein fractions. Earlier, these proteins were considered as sample contaminates and their MT-binding activity was neglected. Recently, their interaction with MTs has shown to be of physiological relevance. In *Arabidopsis*, metabolism-related proteins were predicted to form 21% of the tubulin-interacting fraction (Chuong et al., 2004). Thirteen percent of metabolism-related proteins were detected while searching for MT-interacting proteins exhibiting differential accumulation during tracheary element development (Derbyshire et al., 2015). Nevertheless, only few of these proteins were well-studied. Examples discussed here are glyceraldehyde-3-phosphate-dehydrogenase (GAPDH) (Walsh et al., 1989; Chuong et al., 2004), chaperones Hsp70 and Hsp90 (Freudenreich and Nick, 1998; Ho et al., 2009), plant chaperonin complex CCTε subunit (Nick et al., 2000) and enzyme phospholipase Dδ (PLDδ) (Gardiner et al., 2001).

### GAPDH

**Figure 1:**
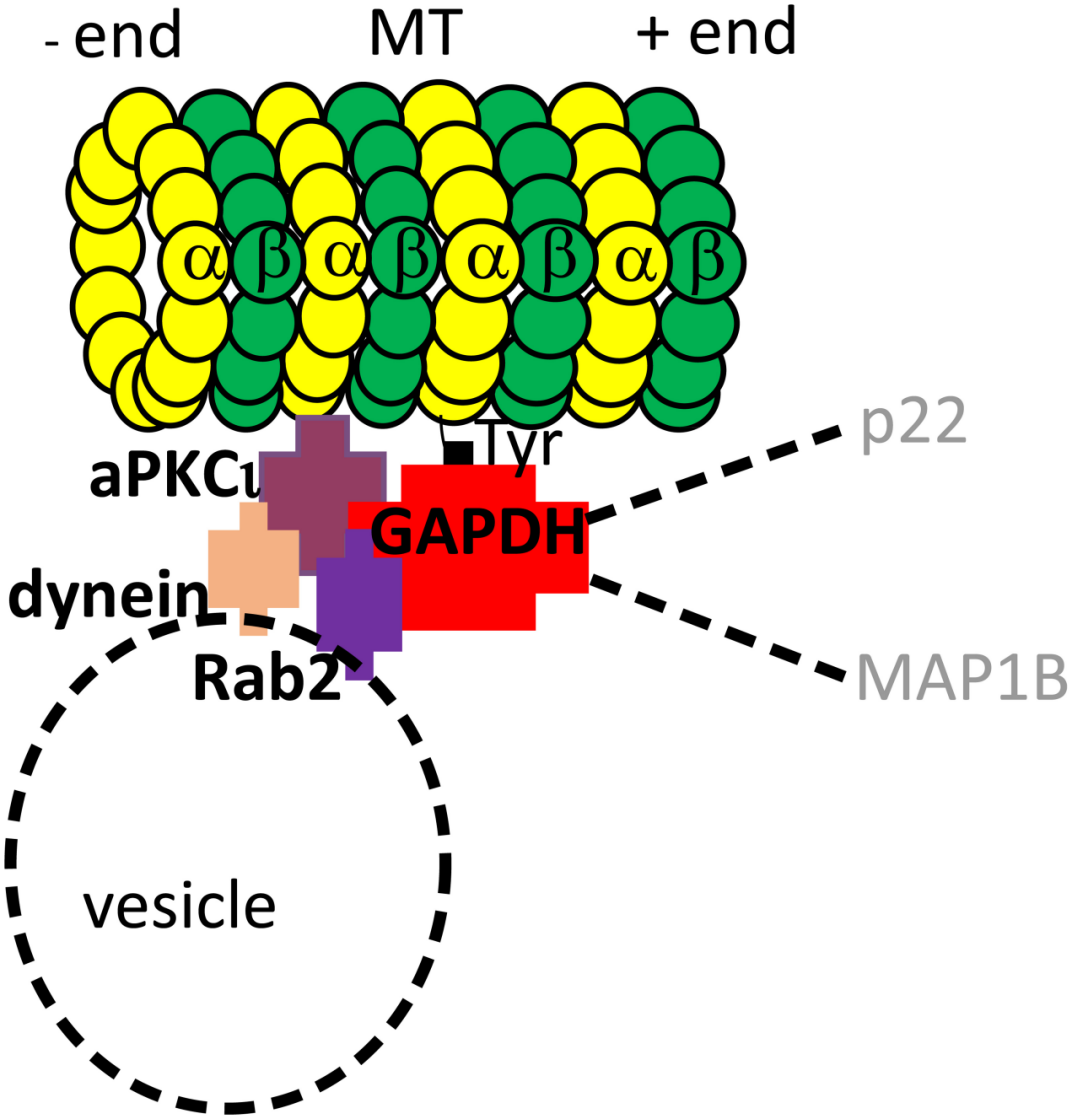
**Model of GAPDH interactions in animal cells**. GAPDH, athough primarily glycolytic enzyme, is an example of a multifunctional MAP that binds to MTs and recruits a multiprotein complex to them. GAPDH binds directly to C-terminus of α-tubulin (Kumagai and Sakai, 1983). Together with PKCι, an atypical protein kinase Cι, GAPDH recruits Rab2GTPase to MTs. Additionally, Rab2GTPase and PKCι recruit dynein motor protein to the complex, which presumably links the complex to vesicle trafficking (Tisdale, 2002; Tisdale et al., 2009). A broader importance of GAPDH complex is suggested by reported binding of other proteins to the complex, such as p22 (Andrade et al., 2004) and MAP1B (Cueille et al., 2007).

GAPDH is a conserved glycolytic enzyme that lyses glyceraldehyde-3-phosphate to 1,3 diphosphoglycerate. GAPDH was the first glycolytic enzyme found to be associated with tubulin and with MTs during polymerization/depolymerization cycles (Kumagai and Sakai, 1983; Somers et al., 1990). It was shown to interact directly with MTs in animals (Kumagai and Sakai, 1983; Walsh et al., 1989; Volker and Knull, 1997; Tisdale et al., 2009). Further, GAPDH plays role in vesicle trafficking, MT array arrangement, DNA replication and repair, nuclear RNA export, apoptosis or stress detection in animals (for review, see Sirover, 1999). GAPDH mediates MT-binding of other MT-interactors, such as Rab2 GTPase, therefore, it physically links MTs and membrane structures involved in secretory pathways of metazoans (Tisdale, 2002; Andrade et al., 2004). RabGTPases further interact with motor proteins that modulate vesicle binding to MTs (Hammer and Wu, 2002; Perez et al., 2002; Howard and Hyman, 2003). Thus, GAPDH represents a multifunctional MAP with the ability to recruit a multiprotein complex to MTs in metazoans (for suggested model, see Figure 1). In plants, GAPDH was found together with other metabolic and protein synthesis enzymes, as well as signaling proteins in the tubulin binding protein fraction, which were isolated from *Arabidopsis* suspension cultures (Chuong et al., 2004). Further, GAPDH interacted with MTs in maize endosperms (Azama et al., 2003). GAPDH was found in the *Arabidopsis* proteomic screen for MT-binding proteins (Derbyshire et al., 2015). However, the physiological role of plant GAPDH interaction with MTs and probable role in multiprotein complex recruitment to MTs remains unknown.

In animals, numerous glycolytic and sucrose metabolism enzymes were found to bind MTs: lactate-dehydrogenase, pyruvate kinase, aldolase, glucose-6-phosphate isomerase, phosphoglycerate-kinase, sucrose synthase, sucrose-UDP glucosyltransferase (Walsh et al., 1989). In plants, enzymes of folate-dependent pathways, fatty acid metabolism, pentose phosphate pathway, phosphate metabolism, amino acid biosynthesis, the tricarboxylic acid cycle, anaerobic glycolysis, and panthothenate biosynthesis enzymes were reported in the tubulin-binding fraction in *Arabidopsis* (Chuong et al., 2004). The significance of these interactions remains to be elucidated, but, as indicated in animals, the interactions of metabolism–related proteins with MTs signify a promising area of discoveries with high biological importance.

### Heat shock protein 90

Heat shock protein 90 (Hsp90) is a highly conserved molecular chaperone essential for protein folding and stability. Along with binding various substrates in animals (Wegele et al., 2004), Hsp90 mediates switches between active and inactive states of regulatory and signaling proteins (Rutherford and Zuker, 1994). In plants, Hsp90 is involved in MAPK cascades (Takabatake et al., 2007) and R-proteins-triggered stress response (Takahashi et al., 2003; Boter et al., 2007). Hsp90 also interacts with actin and tubulin cytoskeleton (Koyasu et al., 1986; Sanchez et al., 1988; Wegele et al., 2004). Due to its numerous substrates and interacting structures including MTs, Hsp90 functions at the interface of several developmental pathways in eukaryotes (Rutherford and Lindquist, 1998).

In animal cells, Hsp90 interaction with MTs appears to be complex; it interacts with tubulin dimers (Sanchez et al., 1988; Weis et al., 2010), with polymerized MTs (Fostinis et al., 1992; Williams and Nelsen, 1997) and, Hsp90 is a subunit of the heterocomplex associated with MTs during the nuclear transport of steroid hormones (Pratt et al., 1999; Harrell et al., 2002). It is found in the centrosome (Lange et al., 2000). Together with other centrosomal proteins, Hsp90 is involved in centrosome assembly and function (de Carcer et al., 2001; Glover, 2005; Basto et al., 2007).

**Figure 2:**
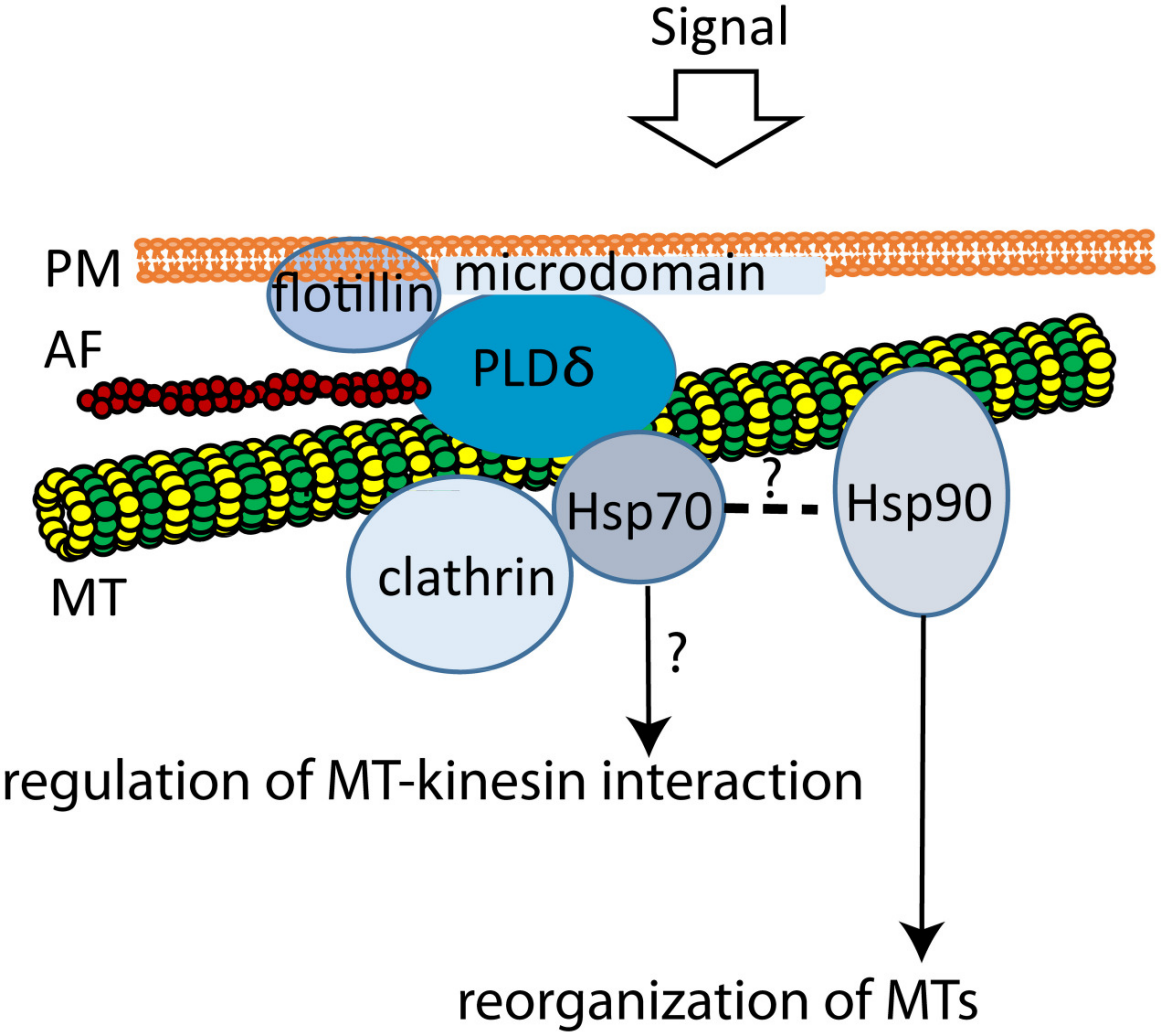
**Model of plant MTs interactions with plasma membrane microdomains**. The multiprotein complex composed of flotillin, PLDδ, MTs, AFs, Hsp70, and clathrin possibly creates the PM-cytoskeleton continuum and thus may be involved in cell signaling transduction and vesicle trafficking. PLDδ links PM with cortical MTs (Marc et al., 1996; Gardiner et al., 2001) at sites where cell signaling processes take place, since it binds to plant flotillin, a microdomain marker (Ho et al., 2009). Actin, tubulin, Hsp70, Hsp90, and clathrin heavy chain are further PLDδ interactors (Ho et al., 2009). Furthermore, plant MTs interact directly with both Hsp70 (Parrotta et al., 2013), and Hsp90 (Krtkova et al., 2012). Assumed heterocomplex chaperone machinery consisting of Hsp70 and Hsp90 (Pratt et al., 2001) may further control the reorganization of MTs (Hsp90, Krtkova et al., 2012) or interaction with kinesins (Hsp70, Parrotta et al., 2013). Model adapted from Ho et al. (2009).

In plants, Hsp90 is known to associate with tubulin dimers, cortical MTs and phragmoplast MTs (Freudenreich and Nick, 1998; Petrasek et al., 1998; Krtkova et al., 2012). Tobacco Hsp90 binds directly to polymerized MTs *in vitro* (Krtkova et al., 2012). Since the inhibition of Hsp90 severely impairs MT re-assembly after cold-induced de-polymerization, Hsp90 interaction with MTs conceivably plays a role in rapid MT re-assembly important during environmental changes or stress (Krtkova et al., 2012; Figure 2).

### The cytosolic chaperonin-containing TCP-1 complex

The cytosolic chaperonin-containing TCP-1 complex (CCT), also known as the TCP1 ring complex (TRiC), plays a role in folding of newly synthetized actin and tubulin molecule and in organization of the MT cytoskeleton in mammalian cells (Lewis et al., 1997). In plant cells, its CCTε subunit localizes along phragmoplast MTs and cortical bundles that accompany secondary-wall thickenings (Nick et al., 2000). It is possible that CCTε is involved in the reorganization of microtubular cytoskeleton by regulating tubulin folding (Moser et al., 2000).

### Heat shock protein 70

Proteins of Hsp70 family are involved in a range of cellular processes, predominantly under stress conditions, such as heat. They prevent protein aggregation, assist in protein refolding, import and translocation, signal transduction and transcriptional activation (for review, see (Zhang and Glaser, 2002; Wang et al., 2004). In the plant cortical region, Hsp70 associates with MTs and tubulin, as well as with PLDδ (Ho et al., 2009). In *Chlamydomonas*, the failure of the Hsp70-Hsp40 chaperone system to recognize or fold the client protein(s) results in increased MT stability and resistance to the MT-destabilizing effect of the herbicides (Silflow et al., 2011). Parrotta et al. (2013) identified a Hsp70 isoform in the pollen tube of tobacco that binds to MTs in an ATP-dependent manner. Interestingly, Hsp70 binding to MTs was also dependent on the binding of a kinesin motor p90 (Parrotta et al., 2013). This raises a possibility that Hsp70 may modulate kinesin action on MTs, a phenomenon observed also in other systems (Terada et al., 2010).

## Protein translation machinery proteins

The interaction of cytoskeleton with polysomes was first identified in the 1970's. Since then, many data supporting the role of actin and MTs in metazoan translation machinery localization and regulation were published (for review see Kim and Coulombe, 2010). Plant transcription machinery seems to interact with the cytoskeleton as well (Muench and Park, 2006). In proteomic screens performed in plants, large groups of proteins interacting with MTs are primarily involved in RNA transcription processes. For example, Chuong et al. (2004) predicted 21% of tubulin-binding proteins assist in RNA binding and 19% in translation. Similarly, in the screen of MT-associated proteins with changed expression during tracheary element differentiation, 13% of isolated proteins were predicted to be involved in protein synthesis, and 19% in DNA or RNA binding (Derbyshire et al., 2015). In a model proposed for plant cells, the predominant role in the transport and localization of translation machinery components is assigned to actin cytoskeleton, whereas MTs may anchor and perhaps influence the translation process (Muench and Park, 2006). Indeed, some proteins participating in the translation are repeatedly reported to associate with MTs or tubulin. Here, elongation factor 1α and THO2 proteins are discussed.

### Elongation factor 1α

Elongation factor 1α (EF1α) is a translational factor that binds aminoacyl-tRNA and ribosomes in a GTP-dependent manner (Carneiro et al., 1999). Additionally, EF1α was reported to bind and to bundle actin filaments (AFs) in animal cells (Murray et al., 1996). It is believed that F-actin-bound EF1α is translationally inactive, since F-actin sequesters elongation factor 1α from the interaction with aminoacyl-tRNA in a pH-dependent reaction (Liu G. et al., 1996). This suggests the role of EF1α binding to F-actin in the regulation of proteosynthesis. In addition to this, this protein was shown to sever MTs (Shiina et al., 1994). EF1α is repeatedly present in plant MAP-enriched protein fractions (for example Durso and Cyr, 1994; Chuong et al., 2004; Hamada et al., 2013; Derbyshire et al., 2015). EF1α influences AF dynamics (Murray et al., 1996) and MT dynamics in Ca^2+^ and calmodulin-dependent manner (Durso and Cyr, 1994; Moore et al., 1998). Since Ca^2+^ and calmodulin are key players in plant cell signaling, EF1α regulation of cytoskeletal dynamics can serve as a manual transmission stick connecting the cytoskeleton and plant developmental and signaling pathways.

### THO2

In animal and yeast cells, THO2 is part of the THO-TREX complex that participates in mRNA metabolism and nuclear export (Koehler and Hurt, 2007). Hamada et al. (2009) described tobacco putative THO2-related protein (NtTHO2) as a MT-associated protein which binds directly to MTs *in vitro* and co-localizes with cortical MTs *in vivo*, indicating its role in translation targeted to specific plant cell compartments.

## Plasma membrane interacting proteins

In plant cells, cortical MTs underlie the plasma membrane (PM) (Dixit and Cyr, 2004). The association of cortical MTs to the PM is related to the guiding of cellulose synthase complexes (CESAs), enzyme complexes in the plasma membrane that synthesize cellulose into the extracellular space (Paredez et al., 2006). Surprisingly, only a subtle number of proteins were proven to mediate the interaction of cortical MTs with the plasma membrane. In this section, phospholipase D and developmentally-regulated plasma membrane polypeptide (DREPP) proteins are discussed. Both were first reported to participate in MT unrelated processes, however, their roles associated with MTs were revealed later. Some plant formins were reported to interact with the PM and MTs as well. Being primarily actin-associated proteins, they are discussed later in a separate chapter.

### Phospholipase D

PLDs with N-terminal lipid binding domain are important signaling enzymes in plant cells (Munnik, 2001; Elias et al., 2002). Various PLD isoforms differ in their affinity to different substrates—membrane phospholipids. These are cleaved by PLDs to produce signaling molecules (Munnik, 2001; Wang, 2002).

Phospholipase D δ (PLDδ) is a central enzyme of phospholipid signaling in plants. It cleaves plasma membrane (PM) phospholipids to produce phosphatidic acid (PA) and predominantly ethanolamine and choline (for review, see Wang, 2002). PLDδ isoform strongly associates with PM (Gardiner et al., 2001; Wang and Wang, 2001) and connects it physically with cortical MTs (Marc et al., 1996; Gardiner et al., 2001). Upon stress, e.g., NaCl, hypoosmotic stress, xylanase or mastoparane treatment, PLDδ is activated and triggers MT reorganization (Dhonukshe et al., 2003). The mechanism of PLD-triggered reorganization is likely based on the activation of PLD on the plasma membrane, which leads to the release of MTs from the membrane and MTs reorientation (Dhonukshe et al., 2003). Another potential mechanism of PLD-based MT reorganization mechanism may involve the role of PLD signaling product, PA, on MT (for review see Pleskot et al., 2014).

The importance of PLDδ in plants is confirmed by the plasma membrane and MT-binding discussed above and its interaction with actin (Ho et al., 2009). Phospholipase Dδ is thus discussed hereinafter as an example of a protein potentially integrating multiple structures into a functional complex in plants.

### PCaP1/MDP25 and PCaP2/MAP18

DREPP (Developmentally-Regulated Plasma membrane Polypeptide) proteins include a family of plant-specific proteins that interact with the plasma membrane (Gantet et al., 1996). *Arabidopsis* DREPP family contains proteins PCaP1 named also MDP25 (Ide et al., 2007; Li et al., 2011), and a divergent paralog PCaP2, first described as a Microtubule-Associated Protein 18 kDa MAP18 (Wang et al., 2007; Kato et al., 2010). PCaP1/MDP25 links calcium signaling to the regulation of cytoskeleton organization. Under normal conditions, PCaP1/MDP25 is localized to the plasma membrane. Increased calcium levels cause PCaP1/MDP25 to partially dissociate from the plasma membrane and to move into the cytosol. In the hypocotyl, cytosolic PCaP1/MDP25 binds and destabilizes cortical MTs by depolymerization and subsequently inhibits hypocotyl cell elongation (Li et al., 2011). In the subapical region of pollen tubes, PCaP1/MDP25 binds directly to actin cytoskeleton and severs individual actin filaments, thus negatively regulating pollen tube growth (Qin et al., 2014). PCaP2, previously reported as MT-binding MAP18 (Wang et al., 2007), is a plant-specific protein found only in *Arabidopsis* that is involved in intracellular signaling in growing root hairs and pollen tubes. PCaP2/MAP18 is localized in plasma membranes possibly via N-myristoylation, and destabilizes MTs (Keech et al., 2010). It is associated with specific PtdInsPs and it exhibits the capacity to bind calcium and calcium–calmodulin (Ca^2+^–CaM) complex (Kato et al., 2010). It is possible that association and/or dissociation of PCaP2/MAP18 with PtdInsPs via oscillation in Ca^2+^ cytosolic concentration regulate the signaling function of PtdIns(4,5)P2, which includes regulation of ion channels (Suh and Hille, 2008), cytoskeletal organization and membrane traffic (Meijer and Munnik, 2003; Lee et al., 2007; Kato et al., 2010).

## Actin binding proteins as multifunctional MAPs

In plants, AFs are crucial for cell polarity, division, membrane trafficking and thus, growth and development. Their organization and dynamics is modulated by actin binding proteins, such as formins, Arp2/3 complex, profilin, cofilin, myosin etc. (for review see Thomas et al., 2009). An increasing list of proteins interacting with both actin and MTs in plants was reported (for review see Petrasek and Schwarzerova, 2009). The existence of proteins interacting with both AF and MT is not surprising, since actin-cytoskeletal functions are fulfilled in a close collaboration with MT cytoskeleton (Collings, 2008; Smertenko et al., 2010; Sampathkumar et al., 2011), e.g., during plant cell division, in PPB and phragmoplast (Traas et al., 1987; Mineyuki, 1999; Sano et al., 2005; Wu and Bezanilla, 2014). The following examples were reported to interact with both AFs and MTs: plant formins (Deeks et al., 2010; Li et al., 2010), a subunit of ARP2/3 protein complex ARPC2 (Havelková et al., 2015), conventional MAPs, such as kinesins (Preuss et al., 2004; Frey et al., 2009; Klotz and Nick, 2012; Schneider and Persson, 2015), plant specific MAPs, such as 190 kDa polypeptide (Igarashi et al., 2000) and SB401 in *Solanaceae* (Huang et al., 2007), enzyme PLDδ (Ho et al., 2009) or protein DREPP/AtPCaP1/MDP25 (Li et al., 2011; Qin et al., 2014). Here, proteins with primary functions related to actin cytoskeleton organization that were found to interact also with MTs (formins and ARPC2) are discussed.

### AtFH4 and AFH14

Formins nucleate actin and contribute to the filament growth, thus, they participate in cell polarity, morphogenesis and division (Sagot et al., 2002; Kovar and Pollard, 2004; Pruyne et al., 2004; Ingouff et al., 2005). However, some plant formins also bind directly to MTs (Deeks et al., 2010; Li et al., 2010). Formins are characterized by the presence of formin homology-2 and formin homology-1 (FH2 and FH1, respectively) domains that are common in mammals and plants (Blanchoin and Staiger, 2010). Besides FH1 and FH2 domains important for actin nucleation, plant AtFH4 contains a plant-specific transmembrane domain, and a specific GOE domain that binds directly to MTs (Deeks et al., 2010). Thus, AtFH4 represents a protein that links both membranes, MTs and AFs in plant cells. Another plant formin called FORMIN14 (AFH14) appeared to bind directly either AF or MTs (Li et al., 2010). Unlike AtFH4, the FH2 domain of AFH14 is critical for both MT and AF binding and bundling. AFH14 localized to typical plant MT structures, such as preprophase band (PPB), spindles, or phragmoplast. MTs competed with AFs to bind AFH14, and the overexpression of AFH14 caused co-alignment of MTs with AFs, which perturbed the progress of cell division (Li et al., 2010). Therefore, actin-binding proteins formin AtFH4 and AFH14 represent multifunctional MAPs with specialized function in mediating AF and MT crosstalk.

### Actin related protein 2/3 complex subunit 2

Arp2/3 complex represents the second mechanism of AF nucleation. This evolutionarily conserved complex of 7 subunits (Welch et al., 1997) is functional also in plants, where it controls several aspects of plant morphogenesis (for review see Deeks and Hussey, 2005; Yanagisawa et al., 2013). Recently, it has been shown that actin related protein 2/3 complex subunit 2 (ARPC2) of Arp2/3 complex binds directly to MTs. It is possible that the ARPC2 subunit of Arp2/3 complex mediates the interaction between MTs and AFs in plants (Havelková et al., 2015). Alternatively, Arp2/3-based interaction of MTs and AFs may contribute to mutual dynamic regulation of AFs and MTs. ARPC2 protein thus, represents another multifunctional MAP with the primary role unrelated to MT binding.

## Multifunctional MAPs involved in signaling crosstalks

Stimuli from the outer environment are transferred into the plant cell across the rigid cellulose cell wall and lipid plasma membrane. Specific receptors on the plasma membrane may transfer stimuli by the cell wall-PM-cortical MT continuum. According to the recent studies, mediators in this physical continuum may be PLDδ and/or plant-specific formins with transmembrane domains. These proteins bind to the plasma membrane, are associated with cortical MTs and actin, and as in the case of AtFH4, possess extracellular extensin-like motifs that can anchor it to the cell wall compounds (Baluska and Hlavacka, 2005; Deeks et al., 2005, 2010; Ho et al., 2009; Cvrckova, 2013). Formins can further mediate attachment of endomembrane compartments, such as the ER or secretory vesicles, to the MT cytoskeleton (Cvrckova et al., 2015).

In addition to binding to PM phospholipids, PLDδ binds to plant flotillin homolog (Ho et al., 2009), a lipid microdomain marker (Martin et al., 2005). Lipid microdomains are PM detergent-resistant regions that are important for the assembly of multimolecular signaling complexes containing G-proteins or kinases (Martin et al., 2005; Dunkley et al., 2006; Tapken and Murphy, 2015). Therefore, PLDδ may link PM with MTs at sites where cell-signaling processes take place. However, PM and MTs are not PLDδ's sole interacting structures. F-actin (Kusner et al., 2003), Actin 7, Hsp70, ATPase and clathrin heavy chain (Ho et al., 2009) were reported as PLDδ interactors, too. Thus, by interacting with both cytoskeletal networks, PLDδ is a possible mediator in the cell wall-PM-cytoskeleton continuum. Its additional interaction with Hsp70 may contribute to the signal transduction to the cytosol (Ho et al., 2009). This interaction is probably mediated by MTs. As authors hypothesize, multiprotein complexes composed of flotillin, PLDδ, MTs, AFs, Hsp70, and clathrin indirectly bound to PLD are involved in cell signaling and vesicle trafficking (Ho et al., 2009).

Hsp70 and Hsp90 cooperate in the signaling, protein folding, stabilization, and turnover by the formation of multichaperone complexes (Pratt et al., 2001, 2010). They have been reported as tubulin interactors as well (Freudenreich and Nick, 1998; Garnier et al., 1998; Petrasek et al., 1998; Parrotta et al., 2013). Since Hsp90 localizes to the cortical MTs and was isolated as the protein interacting with both PM and MTs (Krtkova et al., 2012), it is likely that the whole complex composed of Hsp90, Hsp70, MTs and actin is linked to the lipid microdomain platforms by PLDδ. By this mean, the PM-cytoskeleton continuum involved in cell signaling may be established (Figure 2).

**Figure 3:**
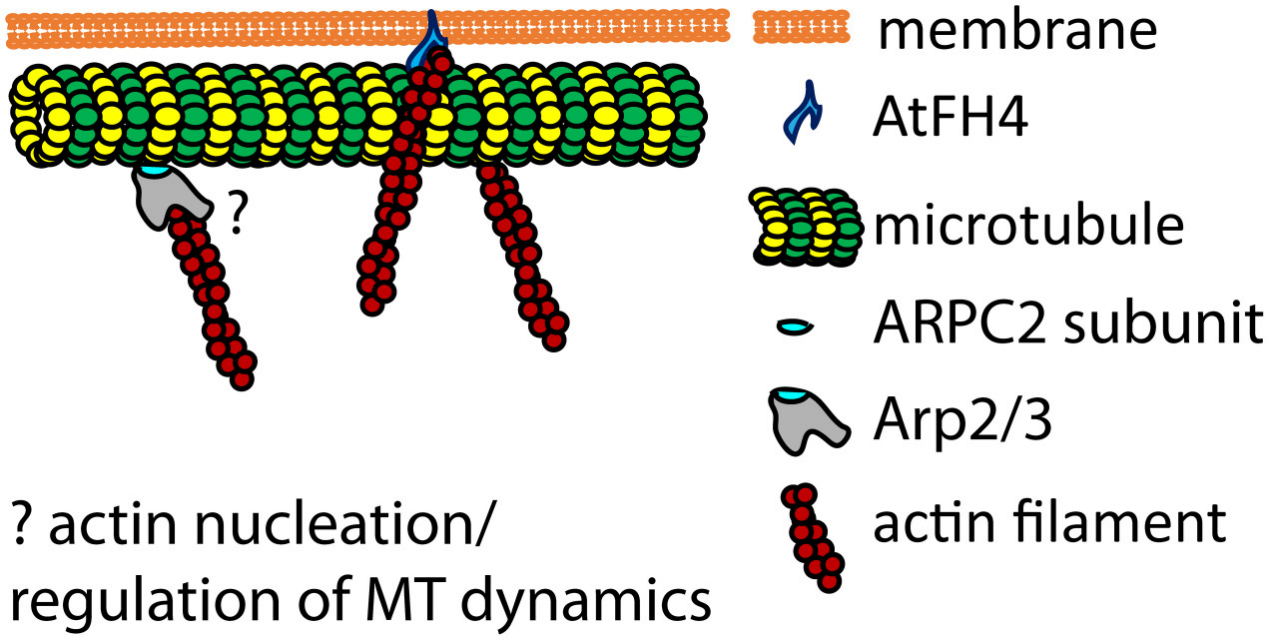
**Model of plant MTs interactions with actin filaments based on actin nucleators**. Formin AtFH4 is anchored in the PM and binds to AFs, providing a supportive scaffold for MTs attachment (Deeks et al., 2010). ARPC2 subunit of Arp2/3 complex binds directly to MTs (Havelková et al., 2015). The role of ARPC2-MTs interaction in the cross-linking of AFs and MTs through Arp2/3 complex, or in the regulation of AFs and MTs dynamics, remains to be elucidated (question mark). Model adapted from Deeks et al. (2010).

Formin interacting with both AFs and MTs (AtFH4) is possibly involved in cell signaling as well. Via its extracellular and transmembrane domain, it may transduce mechanical stimuli from the plant cell wall across PM to both cytoskeletal networks. According to the model for AtFH4 proposed in Deeks et al. (2010; see also Figure 3), mechanical stimuli transduced by formin-mediating PM-cytoskeleton continuum may be preferentially manifested in actin dynamics changes. In this hypothesis, MTs represent structural scaffolds enabling FH2 domain of AtFH4 to perform its actin-nucleating function. AtFH4 also co-aligns MTs with endoplasmic reticulum, suggesting a further role of AtFH4 at the interface of actin and MT cytoskeleton (Deeks et al., 2010).

## Physiological demonstration of multifunctional MAPs-MT interaction—future prospects

Stability, dynamics and organization of MTs is modulated by their interacting proteins. MAPs, such as +TIPs (for review, see e.g., Akhmanova and Steinmetz, 2008) or other structural MAPs (Gardiner, 2013) coordinate MT reorganization events spatiotemporally, thus controlling the localization of MTs in the plant cell during specific environmental or developmental conditions. Such events underlie plant cell shape changes and plant tissue differentiation, determining survival of the plant organism through proper growth regulation.

MTs apparently require further mediating-proteins as well. These mediators may be the traditional motor or structural MAPs, but also proteins with another primary function than MT-binding. These proteins may interact with MTs in short time limits, under specific environmental conditions or interact with MTs weakly or indirectly as members of MT-associated structures or complexes. Some of these proteins were discussed in this review. These spatiotemporally tightly regulated physiological functions, or secondary interactions, as well as functions of single complex subunits, are difficult to detect. Nevertheless, important progress has been made recently in identifying new multifunctional MAPs; new proteins will be added to the list in the future. Plant hormone signaling, stress and pathogen response, development of specific morphological structures and other plant specific processes represent areas for investigating new highly specific MT-associated proteins. Investigating into the functional interactions between MTs and both protein synthesis machinery and metabolism-related enzymes in plants is an exciting research area awaiting deeper exploration.

## Author contributions

All authors listed, have made substantial, direct and intellectual contribution to the work, and approved it for publication.

### Conflict of interest statement

The authors declare that the research was conducted in the absence of any commercial or financial relationships that could be construed as a potential conflict of interest.

## Abbreviations

CCT: cytosolic chaperonin containing TCP-1
EF1α: elongation factor 1α
EF2: elongation factor 2
FH2: formin homology 2
GAPDH: glyceraldehyde-3-phosphate-dehydrogenase
GDA: geldanamycin
MAP: microtubule-associated protein
MDP: microtubule destabilizing protein
MT: microtubule
PA: phosphatidic acid
PcaP: plasma membrane-associated Ca^2+^-binding protein
PLD: phospholipase δ
PPB: preprophase band
+TIP: +tip interacting protein.
